# mTOR Modulates Methamphetamine-Induced Toxicity through Cell Clearing Systems

**DOI:** 10.1155/2018/6124745

**Published:** 2018-10-29

**Authors:** Gloria Lazzeri, Francesca Biagioni, Federica Fulceri, Carla L. Busceti, Maria C. Scavuzzo, Chiara Ippolito, Alessandra Salvetti, Paola Lenzi, Francesco Fornai

**Affiliations:** ^1^Department of Translational Research and New Technologies in Medicine and Surgery, Human Anatomy, University of Pisa, Via Roma 55, Pisa 56126, Italy; ^2^I.R.C.C.S Neuromed, Via Atinense 18, Pozzilli 86077, Italy; ^3^Department of Clinical and Experimental Medicine, University of Pisa, Via Roma 55, Pisa 56126, Italy

## Abstract

Methamphetamine (METH) is abused worldwide, and it represents a threat for public health. METH exposure induces a variety of detrimental effects. In fact, METH produces a number of oxidative species, which lead to lipid peroxidation, protein misfolding, and nuclear damage. Cell clearing pathways such as ubiquitin-proteasome (UP) and autophagy (ATG) are involved in METH-induced oxidative damage. Although these pathways were traditionally considered to operate as separate metabolic systems, recent studies demonstrate their interconnection at the functional and biochemical level. Very recently, the convergence between UP and ATG was evidenced within a single organelle named autophagoproteasome (APP), which is suppressed by mTOR activation. In the present research study, the occurrence of APP during METH toxicity was analyzed. In fact, coimmunoprecipitation indicates a binding between LC3 and P20S particles, which also recruit p62 and alpha-synuclein. The amount of METH-induced toxicity correlates with APP levels. Specific markers for ATG and UP, such as LC3 and P20S in the cytosol, and within METH-induced vacuoles, were measured at different doses and time intervals following METH administration either alone or combined with mTOR modulators. Western blotting, coimmunoprecipitation, light microscopy, confocal microscopy, plain transmission electron microscopy, and immunogold staining were used to document the effects of mTOR modulation on METH toxicity and the merging of UP with ATG markers within APPs. METH-induced cell death is prevented by mTOR inhibition, while it is worsened by mTOR activation, which correlates with the amount of autophagoproteasomes. The present data, which apply to METH toxicity, are also relevant to provide a novel insight into cell clearing pathways to counteract several kinds of oxidative damage.

## 1. Introduction

Methamphetamine (METH) is a highly addictive and neurotoxic drug, which causes a variety of neuropsychiatric alterations mainly affecting the dopamine (DA) mesostriatal and mesolimbic systems in the brain [1, 2]. Exposure to repeated doses of METH produces striatal DA depletion and loss of mesostriatal DA terminals [3–12].

In the cell body of the substantia nigra *pars compacta* (SNpc), METH produces alterations in the cytoplasm which also occur in DA-PC12 cells and extend to the cytoplasm and nucleus of striatal GABA neurons [6, 13–16]. These alterations configure as multilamellar whorl staining for ubiquitin, parkin, and alpha-synuclein [6, 15, 17]. Recent studies indicate that high METH doses may reduce the number of nigral cell bodies [8, 18]. METH toxicity against DA cell bodies and axons relates to an increase of DA release and oxidative species [19]. In fact, METH alters the vesicular storage of DA [20–22], it inhibits physiological DA metabolism, which is naturally operated by MAO-A [23, 24], and it reverts and/or inhibits the activity of the plasma membrane DA transporter (DAT), thus leading to a loss of DAT-binding sites [1, 25, 26]. All these effects contribute to rise of dramatically free DA levels within the cytosol of DA-containing cells. Since DA is no longer metabolized by MAO-A, it undergoes self-oxidation and spontaneous conversion to DA quinones, which in turn generate highly reactive oxidative species [27, 28]. In this way, a redox imbalance is generated by METH, which is detrimental for the integrity of both axon terminals and cell bodies where oxidized proteins, lipids, and nucleic acids are generated [29, 30]. A key molecular mechanism of protein oxidation consists in binding to cysteinyl residues to generate disulphuric bridges, which alter protein conformation [28, 31]. In this way, misfolded proteins such as alpha-synuclein [6, 14], ubiquitin [6, 32], prion protein [33], and parkin [6, 34] are generated. Again, METH inhibits complex II of the mitochondrial respiratory chain, which further elevates oxidative species and increases the number of altered mitochondria [35–39]. METH also oxidizes lipids to produce highly reactive by-products such as 4-hydroxynonenal [34, 40, 41]. All these oxidized substrates represent a target for cell clearing systems, which promote their removal. Thus, autophagy (ATG) and ubiquitin-proteasome (UP) represent a powerful defense to counteract redox imbalance generated by such a drug of abuse, and they are both challenged by METH administration. In detail, UP activity is inhibited by METH [13, 15, 16, 34], while UP inhibitors produce subcellular alterations which overlap with those produced by METH [6, 14, 42]. In line with this, METH toxicity is enhanced by concomitant exposure to UP inhibitors [15, 43]. ATG is quickly engaged during METH in PC12 cells [22, 44] and *in vivo*, in the SNpc and striatum [6, 15, 45]. Similarly to UP inhibitors, ATG blockers worsen METH toxicity [37]. Despite a massive engagement of ATG, which should sort neuroprotection, its activity is impaired by METH itself since the high amount of substrates (misfolded proteins and damaged mitochondria) engulfs this clearing system [16, 37, 46]. Therefore, despite being ATG overexpressed following METH [22, 44], it is not considered to be effective due to a lack of its progression [37, 46]. Such a combined defect in cell clearing systems produced by METH paves the way to deleterious effects induced by oxidative species, which are abundantly produced by such a drug of abuse.

Recently, a cell clearing organelle, which possesses both ATG and UP components, was described. This organelle appears as a multilamellar vacuole, which carries both UP and ATG key antigens [47]. This organelle corresponds to the “autophagoproteasome” (APP) as being defined in the Glossary published in the consensus manuscript “Guidelines for the Use and Interpretation of Assays for Monitoring Autophagy (3rd Edition)” by Klionsky et al. [48]. In the recent manuscript, it was demonstrated that APP is strongly activated by mTOR inhibition [47]. In fact, when the mTOR inhibitor rapamycin is administered, roughly all UP-positive puncta detected by P20S immunostaining at confocal microscopy move towards LC3-positive vacuoles, thus producing a massive switch from cytosolic to compartmentalized proteasome [47]. Despite a strong involvement of UP and ATG per se during METH toxicity, no study so far investigated what happens to this merging organelle. In the present manuscript, we dissect the ultrastructural morphometry of both UP and ATG components in different cell compartments, alone and in combination to merge within the autophagoproteasome (APP), under the effects of various METH doses at different time intervals. A variety of techniques were used to investigate these effects encompassing plain light microscopy, confocal microscopy, transmission electron microscopy, Western blotting, and coimmunoprecipitation. In detail, we aimed to assess whether (i) the autophagoproteasome was operating in the DA-containing PC12 cell line, (ii) the autophagoproteasome was modified following METH exposure, (iii) the amount of this organelle was associated with the modulation of METH toxicity, and (iv) whether these phenomena depend on mTOR activity as tested during either mTOR inhibition or activation.

## 2. Materials and Methods

### 2.1. Cell Cultures

In the current study, we chose the rat pheochromocytoma PC12 cell line, since these cells are able to synthetize and release DA and they express DA receptors on their external membrane. This is key in the case of METH, which exerts its mechanisms of action mainly by affecting molecular targets, which regulate DA transmission. In fact, the presence of DA and DA receptors, as well as DA uptake mechanisms, renders PC12 cell lines closer to DA terminals compared with their ancestors (i.e., chromaffin cells of the adrenal medulla). This concept is reinforced by the presence of the isoform of monoamine oxidase (MAO) type A, which characterizes also DA neurons, contrasting with the established prevalence of MAO type B within chromaffin cells of the adrenal medulla. Therefore, PC12 cells represent a model to predict the neurotoxicity of METH on central DA neurons with significant implications for the treatment of neuropsychiatric and neurodegenerative disorders [49].

The PC12 cell line was obtained from a cell bank (IRCCS San Martino Institute, Genova). The cells were grown in RPMI 1640 medium (Sigma-Aldrich, St. Louis, MO, USA) supplemented with heat inactivated 10% horse serum (HS, Sigma) and 5% fetal bovine serum (FBS, Sigma), penicillin (50 IU/ml)/streptomycin (50 mg/ml, Sigma), under standard culture conditions in a humidified atmosphere containing 5% CO_2_ at 37°C. Experiments took place during the log phase of cell growth. At this time, cells were seeded into cell culture plates and they were incubated at 37°C in 5% CO_2_ for further 24 hours. In particular, for transmission electron microscopy (TEM) and coimmunoprecipitation experiments, 1 × 10^6^ PC12 cells were seeded in culture dishes in a final volume of 5 ml. For Western blotting, 5 × 10^5^ cells were seeded in six-well plates in a final volume of 2 ml/well. Finally, for confocal microscopy experiments, 5 × 10^4^ cells were grown on polylysine slides placed in 24-well plates at a final volume of 1 ml/well.

In order to study METH-induced toxicity, PC12 cells were treated with different doses of METH (1 nM, 10 nM, 100 nM, 1 *μ*M, and 10 *μ*M) for 72 hours. In a second set of experiments, PC12 cells were treated with 1 *μ*M or 10 *μ*M of METH for different time exposures (12, 24, and 72 hours). A study from Melega et al. [50] reports data deriving from an i.v. intake of 1000 mg/day METH in humans, approximately corresponding to 10 mg/kg, which can produce neurotoxicity (i.e., loss of DA terminals and neurons) in mice. However, since the present study was designed to assess the ultrastructural effects of METH on specific subcellular organelles, apart from neurotoxicity, we chose METH doses from 1 *μ*M to 10 *μ*M based on the previous studies [6, 15, 37]. In our hands, at doses between 10 and 100 *μ*M of METH in PC12 cell lines, only a few cells survive, and this is further exacerbated by METH doses above 100 *μ*M [6, 43]. This is also due to intrinsic vulnerability of PC12 cells to DA-increasing agents, which explains such a discrepancy with DA neurons [49]. In fact, PC12 cells possess inherent features, which render these cells particularly sensitive to high doses of DA. These include (i) the presence of VMAT-1, which is less specific for the vesicular uptake of catecholamines when compared with its homolog VMAT-2 expressed in the brain, and (ii) low levels of the DAT, thus reduced cytosolic reuptake of DA. Thus, these cells have a limited ability to adapt the neurotransmitter synthesis and vesicle trafficking/release to the synaptic needs, which contrasts with the flexibility of DA neurons to respond appropriately to a releasing stimulus.

Further experiments were carried out to evaluate the effects produced on METH toxicity and APP components by the modulation of mTOR activity. In these experiments, cells were exposed to 100 nM rapamycin and 50 mM asparagine, alone or in combination with 10 *μ*M METH, for 72 hours. When it was combined with METH, rapamycin was added 2 h before METH, while asparagine was administered 30 min before METH. The doses of asparagine and rapamycin were selected based on the previous papers [32, 47]. However, to validate these doses in these experimental conditions, the inhibition or activation of mTOR activity for each compound was tested by measuring the downstream product pS6.

METH and asparagine were dissolved directly in the culture medium. Dilutions of rapamycin were obtained by a stock solution (1 mM of rapamycin dissolved in the culture medium containing 10% DMSO).

### 2.2. Transmission Electron Microscopy

PC12 cells were centrifuged at 1000*g* for 5 min. After removal of the supernatant, the pellet was rinsed in PBS before being fixed. The fixing procedure was carried out with a solution containing 2.0% paraformaldehyde and 0.1% glutaraldehyde in 0.1 M PBS (pH 7.4) for 90 min at 4°C. This aldehyde concentration minimally covers antigen epitopes, while fairly preserving tissue architecture. After removal of the fixing solution, specimens were postfixed in 1% OsO_4_ for 1 h at 4°C; they were dehydrated in ethanol and finally embedded in epoxy resin.

For ultrastructural morphometry, grids containing nonserial ultrathin sections (40–50 nm thick) were examined at TEM, at a magnification of 8000x. Several grids were analyzed in order to count a total number of 50–100 cells for each experimental group. In particular, when counting cell death, 50 cells per group were sampled, while 50 cells per group were sampled to carry out ultrastructural morphometry and immunogold counts; when counting APP, 100 cells per group were used. Each count was repeated at least 3 times by three blind observers.

Plain TEM was implemented by a postembedding immunocytochemistry procedure for antibodies against LC3 and P20S, which were used as markers of ATG and UP pathways, respectively. Antibody specificity was assessed by a number of studies which were partially reported in Table 1 (extramural evidence), and they were routinely used for at least 10 years in our lab (intramural evidence) [51–76].

It is worth mentioning that LC3 and P20S antigens were chosen as markers of ATG vacuoles (LC3 alone) or APP vacuoles (LC3 combined with P20S) accordingly to the manuscript “Guidelines for the Use and Interpretation of Assays for Monitoring Autophagy (3rd Edition)” [48].

Sometimes, in order to validate the count for ATG vacuoles, beclin 1 was used instead of or in combination with LC3 for detecting early time points. No significant difference between LC3- and beclin 1-based counts was detected; thus, results fully express the amount of LC3. At the end of the plain TEM or immunocytochemistry procedure, ultrathin sections were stained with uranyl acetate and lead citrate, and they were finally examined using a JEOL JEM-100SX transmission electron microscope (JEOL, Tokyo, Japan).

#### 2.2.1. Postembedding Immunocytochemistry

Fixing and postfixing solutions and the use of epoxy resin were validated in our previous studies for immunogold-based ultrastructural morphometry [47]. In fact, a combination of aldehydes, OsO_4_, and epoxy resin allows a minimal epitope covering, while preserving cell ultrastructure [47, 77, 78]. In particular, OsO_4_ binds to cell membranes, thus enhancing the contrast of cytosolic compartments, and it prevents the formation of membrane's artifacts, which may mimic vacuoles. Moreover, epoxy resin is advantageous over acrylic resin in preserving cell morphology.

Postembedding procedure was carried out on ultrathin sections collected on nickel grids, which were incubated on droplets of aqueous sodium metaperiodate (NaIO_4_), for 30 min, at room temperature in order to remove OsO_4_. NaIO_4_ is an oxidizing agent allowing a closer contact between antibodies and antigens by removing OsO_4_ [77]. This step improves the visualization of immunogold particles specifically located within a sharp context of cell integrity, and it allows the counting of molecules within specific cell compartments. Then, grids were washed in PBS and incubated in a blocking solution containing 10% goat serum and 0.2% saponin for 20 min, at room temperature. Grids were then incubated with the primary antibody solution containing both rabbit anti-LC3 (Abcam, Cambridge, UK, diluted 1 : 50) and mouse anti-P20S (Abcam, Cambridge, UK, diluted 1 : 50), with 0.2% saponin and 1% goat serum in a humidified chamber overnight, at 4°C. After washing in PBS, grids were incubated with the secondary antibodies conjugated with gold particles (10 nm mean diameter, for gold particle anti-rabbit; 20 nm mean diameter, for gold particle anti-mouse, BB International), diluted 1 : 30 in PBS containing 0.2% saponin and 1% goat serum for 1 h, at room temperature. Control sections were incubated with the secondary antibody only. After washing in PBS, grids were incubated on droplets of 1% glutaraldehyde for 3 min; additional extensive washing of grids on droplets of distilled water was carried out to remove extensive salt traces and prevent precipitation of uranyl acetate.

#### 2.2.2. Ultrastructural Morphometry

In order to distinguish vacuoles (ATG from APP) and to count immunogold particles (ranging from 10 nm to 20 nm), observations were performed directly at TEM at a magnification of 8000x [79] since this represents the minimal magnification at which immunogold particles and all cell organelles can be concomitantly identified.

We started to count from a grid square corner in order to scan the whole cell pellet within that grid square, which was randomly identified. Assessments of vacuoles and measurement of immunogold particles were carried out according to Lenzi et al. [47].

Briefly, we counted the number of unstained vacuoles per cell as vacuoles with single, double, or multiple membranes possessing the same electron density of the surrounding cytoplasm or partly containing some electron dense structure. In each cell, we counted the total number of immunogold anti-LC3 and/or anti-P20S particles placed either in the cytoplasm or within vacuoles and we expressed the number of immunogold particles as the mean per cell. Finally, we counted the number of APPs per cell as a single, double, and multiple membrane vacuoles, in which immunogold particles of LC3 (10 nm) and P20S (20 nm) were colocalized.

### 2.3. Light Microscopy

For light microscopy, PC12 cells were harvested and centrifuged at 800*g* for 5 min to obtain a pellet, which was further resuspended in 0.5 ml of the culture medium in order to obtain a dense cell suspension. This was layered on glass slide spinning at 15,000*g* for 10 min by cytospin (Cytospin 4, Thermo Fisher).

#### 2.3.1. Haematoxylin and Eosin Staining and Cell Count

Cells were fixed with 4% paraformaldehyde in PBS for 15 min and plunged in PBS and then in haematoxylin solution (Sigma) for 20 min. Haematoxylin staining was stopped by washing in distilled water and followed by plunging cells in the eosin solution (Sigma) for a few min. After repeated washing to remove the excess of dye, cells were dehydrated in increasing alcohol solutions, clarified in xylene, and finally covered with the DPX mounting medium (Sigma). Cell count was performed at light microscopy at 40x magnification. Briefly, for each experimental group, the number of stained cells detectable after each specific treatment was counted and expressed as a percentage of the control group. These values represent the means of six independent cell counts.

Moreover, we counted the number of giant cells occasionally observed after 10 *μ*M METH. We considered as giant cells those owning a diameter higher than 14–15 *μ*m. The amount of giant cells out of the total number of cells counted on the glass slide was expressed as a percentage, for each experimental group. The values represent the means of six independent cell counts.

#### 2.3.2. Trypan Blue

For trypan blue staining, PC12 cells were seeded at a density of 1 × 10^4^ cells/well and they were preincubated for 24 h. After METH treatments, PC12 cells were collected and centrifuged at 800*g* for 5 min. The cell pellet was suspended in the culture medium, and 25 *μ*l of cell suspension was added to a solution of 1% trypan blue (62.5 *μ*l) and PBS (37.5 *μ*l). Cells were then incubated for 10 min, at room temperature. Soon after, 10 *μ*l aliquot of this solution was counted at light microscopy using a Bürker glass chamber. Viable and nonviable cells were counted, and cell death was expressed as percentage of trypan blue frankly positive cells out of the total cells. The values represent the means of three independent cell counts.

### 2.4. Confocal Microscopy

PC12 cells were washed in PBS and fixed with paraformaldehyde 4% for 5 min at room temperature. Antigen retrieval was carried out in 100 mM Tris-HCl, 5% urea at 95°C, for 10 min. After washing in PBS, cells were permeabilized in 0.2% Triton X-100, for 10 min. They were blocked in PBS containing 0.1% Tween-20, supplemented with 1% bovine serum albumin (BSA) and 23 mg/ml of glycine, for 30 min. Afterwards, cells were incubated overnight at 4°C in 1% BSA in PBS-T containing 1 : 50 anti-LC3 antibody (Abcam) and 1 : 30 anti-P20S (Abcam). Finally, cells were incubated for 1 h with fluorophore-conjugated secondary antibodies (1 : 200; goat anti-rabbit Alexa 488 and goat anti-mouse Alexa 594, Molecular Probes, Life Technologies) in 1% BSA in PBS-T at room temperature. Then, cells were washed in PBS, and they were mounted in ProLong Diamond Antifade Mountant (Molecular Probes, Life Technologies). The analysis was performed using a Leica TCSSP5 confocal laser scanning microscope (Leica Microsystems, Mannheim, Germany) using a sequential scanning procedure. Confocal images were collected every 400 nm intervals through the *z*-axis of each section by means of 63x oil lenses. Z-stacks of serial optical planes were analyzed using the Multicolor Package software (Leica Microsystems). Negative controls were carried out by omitting primary antibodies.

### 2.5. Coimmunoprecipitation Assay

PC12 cells were homogenized at 4°C in an ice-cold lysis buffer. One microliter of homogenates was used for protein determinations. 30 *μ*g of proteins from whole cell lysates was loaded to perform Western blotting before coimmunoprecipitation. *β*-Actin was used as a loading control for protein levels from the whole cell lysates, on which immunoprecipitation of LC3 was then performed.

Proteins (800 *μ*g) were incubated at 4°C overnight with primary rabbit anti-LC3 antibody (2 *μ*g for each sample; Sigma-Aldrich, Milan, Italy). The antibody/antigen complex was pulled out of the sample using protein A-Sepharose beads. This process isolated the protein of interest from the rest of the sample. Proteins were separated on sodium dodecyl sulphate-polyacrylamide gel (12%) and transferred on immuno-PVDF membrane (Bio-Rad, Milan, Italy) for 1 h. Filter was blocked for 1 h in Tween-20 Tris-buffered saline (TTBS) (100 mM Tris-HCl, 0.9% NaCl, 1% Tween 20, pH 7.4) containing 5% nonfat dry milk. Blot was incubated at 4°C overnight with mouse monoclonal primary antibody anti-P20S (1 : 100, Abcam), mouse monoclonal anti-alpha-synuclein (1 : 1000, BD Biosciences), and rabbit monoclonal anti-SQSTM1 (anti-p62, 1 : 1000, Abcam, Milan, Italy); it was washed 3 times with the TTBS buffer and then incubated for 1 h with secondary peroxidase-coupled antibody (anti-mouse, 1 : 7000; anti-rabbit, 1 : 7000; Calbiochem, Milan, Italy). Then, blot was stripped with a solution of distilled water and 3.5% acetic acid in the presence of 1% NaCl 5 M. Blot was kept in this solution for 20 min, and then, it was washed in TTBS (8 washes for 5 min). Finally, to verify the correct immunoprecipitation, blot was incubated with primary rabbit anti-LC3 antibody (1 : 6000, Sigma-Aldrich), for 1 h, at room temperature. Filter was washed 3 times with the TTBS buffer and then incubated for 1 h with secondary peroxidase-coupled antibody (anti-rabbit, 1 : 7000; Calbiochem, Milan, Italy). Immunostaining was revealed by enhanced chemiluminescence (GE Healthcare, Milan, Italy). The total amount of proteins measured through optical density was normalized for total *β*-actin, which was measured in whole cell lysates, since *β*-actin is not present in LC3 immunoprecipitates. Thus, readers should consider that such a normalization could not refer to the immunoprecipitated blotted proteins, but rather to the total amount of proteins in the very same cells used to carry out the immunoprecipitate.

### 2.6. Western Blotting

PC12 cells were lysed in a buffer (100 mM Tris-HCl, pH 7.5, 5 M NaCl, 0.5 m EDTA, 10% SDS, 1% NP40, IGEPAL), containing protease and phosphatase inhibitor, and centrifuged at 15,000*g* for 20 min at 4°C. The supernatant was collected, and protein concentration was determined using a protein assay kit (Sigma). Samples containing 40 *μ*g of total proteins were solubilized and electrophoresed on a 12% sodium dodecyl sulphate- (SDS-) polyacrylamide gel. Following electrophoresis, proteins were transferred to the nitrocellulose membrane (Bio-Rad Laboratories, MI, Italy). The membrane was immersed in a blocking solution (3% nonfat dried milk in 20 mM Tris and 137 mM NaCl at pH 7.6 containing 0.05% Tween-20) for 2 h on a plate shaker. Subsequently, the membrane was incubated with mouse anti-pS6 primary antibody (1 : 2000; Millipore, Burlington, MA, USA) overnight at 4°C on the plate shaker. Blot was probed with horseradish peroxidase-labeled secondary antibodies, and the bands were visualized with enhanced chemiluminescence reagents (Bio-Rad Laboratories). Image analysis was carried out by ChemiDoc System (Bio-Rad Laboratories).

### 2.7. Statistics

For ultrastructural morphometry data were given as an absolute number concerning the following measurements: (i) unstained vacuoles, (ii) LC3-positive vacuoles, (iii) P20S-positive vacuoles, (iv) LC3 + P20S-positive vacuoles (APP), and (v) immunogold particles (including LC3 and P20S). Ratios were used to express (i) the number of LC3 immunogold particles within vacuoles out of the number of cytoplasmic LC3 immunogold particles and (ii) the number of P20S immunogold particles within vacuoles out of the number of cytoplasmic P20S immunogold particles. All data were reported as the means ± SEM per cell from 50 cells per group in all counts but the LC3 + P20S which was expressed as the means ± SEM from 100 cells per group.

Data on the amount of cell death were expressed as the percentage of the mean ± SEM dead cells out of the total cell number in each grid being analyzed (i.e., 5 total grids, each containing at least 10 cells, for a total of 50 cells for each experimental group).

For confocal microscopy, the amount of P20S + LC3 puncta was counted. Values were expressed as the mean number of puncta ± SEM per cell counted in each slide in two separate experiments (each one carried out in duplicate).

For Western blot optical density was expressed as the means ± SEM calculated in six separate experiments.

All statistical analyses were carried out by using one-way analysis of variance, ANOVA, followed by Sheffè's post hoc analysis. Null hypothesis (H0) was rejected for *p* ≤ 0.05.

## 3. Results and Discussion

### 3.1. Dose and Time Dependencies of METH-Induced Unstained Vacuoles

**Figure 1:**
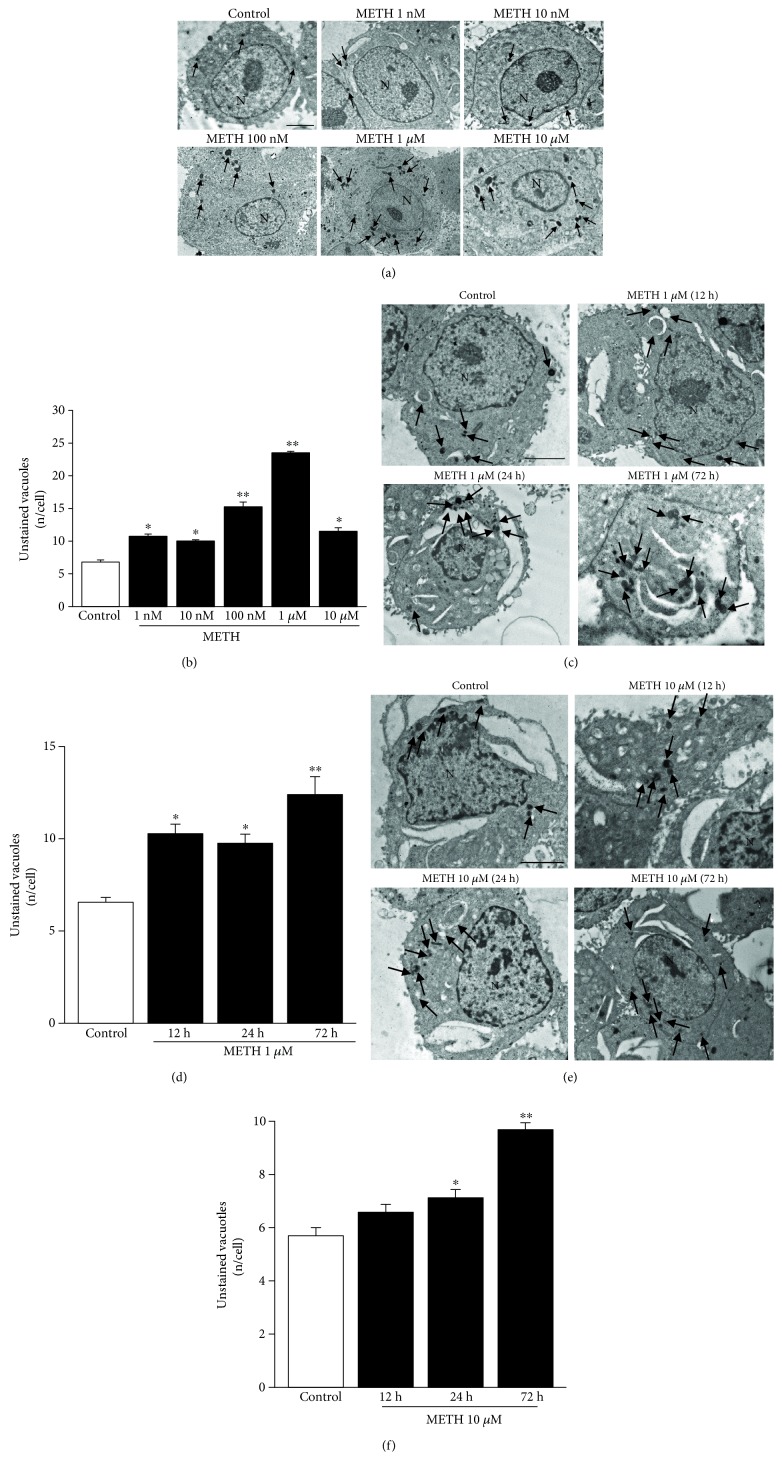
METH increases the amount of unstained vacuoles dose- and time-dependently. (a) Dose-dependent representative pictures of unstained vacuoles (arrows) of control and METH at 72 h treated cells at different doses. (b) Dose-dependent graph of unstained vacuoles per cell at 72 h. (c) Time-dependent representative pictures of unstained vacuoles (arrows) of control and 1 *μ*M METH-treated cells. (d) Time-dependent graph of unstained vacuoles per cell of control and 1 *μ*M METH-treated cells. (e) Time-dependent representative pictures of unstained vacuoles (arrows) of control and 10 *μ*M METH-treated cells. (f) Time-dependent graph of unstained vacuoles per cell of control and 10 *μ*M METH-treated cells. Values are given as the mean number of unstained vacuoles, which were counted in 50 cells per group. Error bars represent the standard error of the mean. ^∗^ *p* ≤ 0.05 vs. control; ^∗∗^ *p* ≤ 0.05 vs. other groups. N = nucleus. Scale bar = 1 *μ*m.

Confirming previous data, METH administration for 72 h filled catecholamine cells with vacuoles, as reported in representative micrographs (Figure 1(a)) and counted in the graph of Figure 1(b). As measured in Figure 1(b), unstained vacuoles increase dose-dependently within a wide range of METH doses (from 1 nM up to 1 *μ*M). At the dose of 1 *μ*M, the number of METH-induced unstained vacuoles reached the peak. Whereas, for the highest dose of METH (10 *μ*M), the number of unstained vacuoles dropped down to levels measured following low METH doses (1 nM and 10 nM). This suggests that at 10 *μ*M METH dose, toxicity occludes the development of novel intracellular structures, even in spared cells. Therefore, the doses of METH 1 *μ*M and 10 *μ*M were chosen for the time dependence study. As reported in representative micrographs of Figure 1(c), a time-dependent increase of unstained vacuoles was produced by METH at the 1 *μ*M dose from 12 h up to 72 h. These effects were evidenced by staining with arrows the unstained vacuoles in each experimental condition to relate representative pictures to counts reported in the graph in Figure 1(d). As expected, even the dose of 10 *μ*M METH at 72 h time-dependently increases the number of unstained vacuoles (Figure 1(f)). This was evident in representative micrographs of Figure 1(e); we investigated the effects of such a METH dose at earlier time intervals (representative pictures of Figure 1(e)). These effects were evidenced by staining with arrows the unstained vacuoles at each time interval to relate representative pictures to counts reported in the graph in Figure 1(f). The number of unstained vacuoles is consistent with the time course and dose dependency of multilamellar bodies produced by METH, which we previously described under a different name (whorls) in this cell line [6].

### 3.2. Dose and Time Dependencies of METH-Induced Cell Death

**Figure 2:**
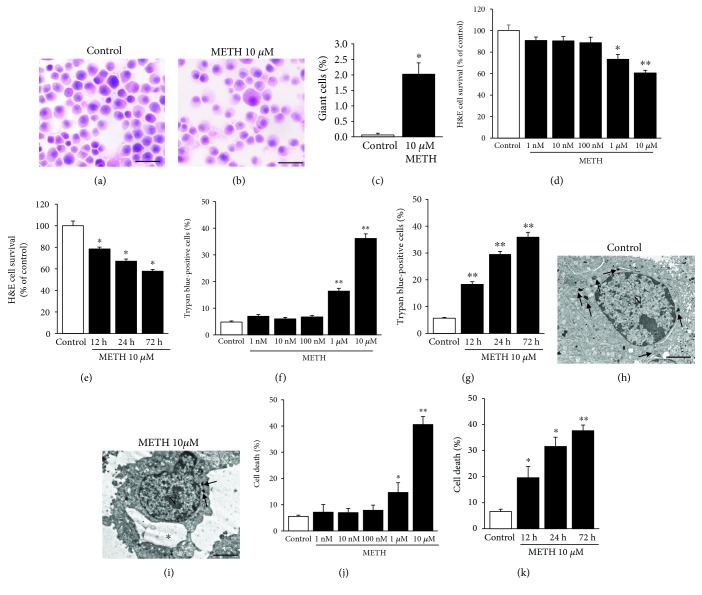
METH induces cell death time- and dose-dependently with a maximal effect at the 1 *μ*M and 10 *μ*M doses. (a) Representative H&E-stained picture from controls. (b) Representative H&E-stained picture following 10 *μ*M METH at 72 h. (c) Graph reporting the percentage of giant cells counted in H&E-stained total cells from controls and METH at 72 h. (d) Dose-dependent graph of H&E-stained cells from control and METH at 72 h. (e) Time-dependent graph of H&E-stained cells from control and 10 *μ*M METH-treated cells. (f) Dose-dependent graph of trypan blue-stained cells from control and METH at 72 h. (g) Time-dependent graph of trypan blue-stained cells from control and 10 *μ*M METH-treated cells. (h) Representative micrograph from a control cell. (h) Representative micrograph from a control cell. (i) Representative micrograph from a METH cell at 72 h. (j) Dose-dependent graph of cell death from control and METH at 72 h. (m) Time-dependent graph of cell death from control and 10 *μ*M METH-treated cells. For the graphs in (c)–(g), values are given as the percentage of cell counted in two triplicates (*n* = 6). For the graphs in (j) and (k), values are given as the percentage of cell counted on 5 grids. Error bars represent the standard error of the mean. ^∗^ *p* ≤ 0.05 vs. control, ^∗∗^ *p* ≤ 0.05 vs. other groups. Arrows point to vacuoles; asterisk (^∗^) indicates a large vacuole. N = nucleus. Scale bar = 23.4 *μ*m (a, b) and 2 *μ*m (h, i).

When assessing the effects produced by a 10 *μ*M dose of METH, there was a dramatic increase (roughly by half) in the amount of cell loss compared with controls and occasionally, giant cells appeared, which were never observed in controls (representative H&E staining of Figures 2(a) and 2(b); graph of Figure 2(c)). The counts for surviving cells carried out at H&E staining revealed a dose- and time-dependent decrease in cell survival (graphs of Figures 2(d) and 2(e), respectively). This was dramatic at 72 h following 10 *μ*M METH. These same results were reproduced by trypan blue-positive counts for dying cells, which confirmed a dose- and time-dependent increase in dying cells (graphs of Figures 2(f) and 2(g), respectively). A similar phenomenon (cell death in the same range of doses and times induced by METH administration) was detected at TEM (representative TEM micrographs of Figures 2(g) and 2(h), respectively). The count of dying cells (either necrotic or apoptotic) at TEM for METH and controls (Figures 2(h) and 2(i), respectively) was overlapping with that reported for trypan blue staining. Remarkably dying cells were higher than controls also following the dose of 1 *μ*M (at 72 h, Figures 2(j) and 2(k)). The pronounced toxicity induced by 10 *μ*M METH is likely to impair cell metabolism even in spared cells, which when analyzed at 72 h own much less vacuoles compared with other doses.

### 3.3. METH Alters Dose and Time Dependency of the Amount and Placement of LC3 Particles

**Figure 3:**
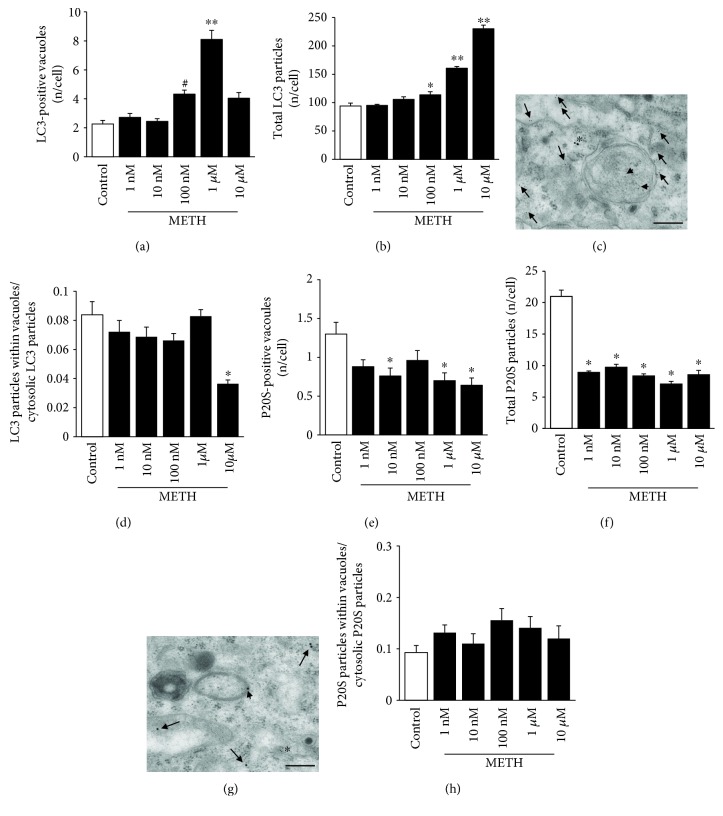
METH alters the amount and placement of LC3 and P20S particles dose-dependently, with P20S being more sensitive than LC3. (a) Dose-dependent graph of the number of LC3-positive vacuoles per cell from controls and METH at 72 h. (b) Dose-dependent graph of total LC3 particles per cell from controls and METH at 72 h. (c) Representative micrograph of LC3-positive vacuole from 10 *μ*M METH at 72 h. (d) Dose-dependent graph of the ratio between the numbers of LC3 particles within vacuoles with respect to cytosolic LC3 particles from controls and METH at 72 h. (e) Dose-dependent graph of the number of P20S-positive vacuoles per cell from controls and METH at 72 h. (f) Dose-dependent graph of total P20S particles per cell from controls and METH at 72 h. (g) Representative micrograph of P20S-positive vacuoles from 10 *μ*M METH at 72 h. (h) Dose-dependent graph of the ratio between the numbers of P20S particles within vacuoles with respect to cytosolic P20S particles from controls and METH at 72 h. Values are given as the mean number of LC3 or P20S particles and vacuoles counted in 50 cells per group. Error bars represent the standard error of the mean. ^∗^ *p* ≤ 0.05 vs. control; ^∗∗^ *p* ≤ 0.05 vs. other groups; ^#^ *p* ≤ 0.05 vs. control and 1 nM and 10 nM METH. Arrows point to free cytosolic LC3 (10 nm) or P20S (20 nm); arrowheads point to LC3 (10 nm) or P20S (20 nm) within vacuoles; asterisk (^∗^) indicates P20S in the cytosol (c) and LC3 in the cytosol (g). Scale bar = 200 nm.

In order to identify ATG and UP components within METH-treated cells, we carried out ultrastructural morphometry by using 10 nm immunogold particles to reveal LC3, while 20 nm immunogold particles were used to stain P20S. Following METH administration, an increase in LC3-stained vacuoles was detected starting at the dose of 100 nM METH, while no increase compared with controls was counted in a lower range of doses (between 1 nM and 10 nM, Figure 3(a)). This was quite unexpected since the count of unstained vacuoles provided in Figure 1(b) indicates a significant increase (almost two-fold) compared with controls even at the dose of 1 nM METH. This is a key point, since unstained vacuoles are considered to correspond to pure ATG vacuoles. Thus, one would expect an overlapping between unstained and LC3-positive vacuoles. Such a discrepancy leaves the issue open on which the nature of METH-induced unstained vacuoles might be. In fact, these vacuoles were induced by METH administration since they increased two-fold compared with controls at the dose of 1 nM and 10 nM METH.

A lack of LC3 staining in these vacuoles for low METH doses suggests that these may not correspond to authentic ATG vacuoles, although they increase two-fold with respect to controls. Although the nature of these unstained vacuoles remains to be defined, the possibility exists that LC3 particles moving within ATG vacuoles remain undetected for these low METH doses. However, as shown in the graph of Figure 3(b), total LC3 particles in the cell for 1 nM and 10 nM METH do not increase either. This indicates a lack of ATG induction for low METH doses. Another possibility deals with the dynamics of ATG vacuoles, which could maturate before LC3 is increased. This hypothesis remains unlikely, since other markers, such as beclin 1, which stains ATG vacuoles earlier than LC3, do not provide any staining either. Moreover, LC3 staining is a gold standard to define autophagosomes, and 72 h should be enough to complete the process. Instead, even at this time interval for low (1 nM and 10 nM) METH doses, LC3 particles do not increase in any cell compartments including the cytosol. This suggests that 1 nM and 10 nM METH do not really increase ATG. Therefore, unstained vacuoles, which minimally occur in control cells and selectively increase following very low METH doses, are likely to belong to other pathways (such as the exosomal compartment). This hypothesis is currently under investigation in the lab. Vacuolar compartments other than ATG may be recruited for low METH doses. It is likely that DA turnover promoted by METH increases vesicle recycling, which may account for these unstained vacuoles. On the other hand, the lowest effects of METH on membrane trafficking may affect other compartments such as retromers or exosomes, which are more bound to cell release than ATG activation.

The increase in vacuoles measured for doses above 10 nM corresponds to LC3-positive (ATG) vacuoles. Data on the amount of LC3-positive vacuoles (shown in representative Figure 3(c)) parallels data on LC3 particles reported in the graph of Figure 3(b). In fact, they increase significantly only at the dose of 100 nM, while they do not differ from controls at low METH doses (1 nM and 10 nM METH). Thus, METH increases LC3 particles (Figure 3(b)) and LC3-positive vacuoles (Figure 3(a)) only at doses higher than 10 nM, although no compartmentalization of LC3 within vacuoles is produced by any dose of METH (graph of Figure 3(d)). In contrast, the trend indicates a dispersion rather than a polarization of LC3 particles from cell vacuoles towards the cytosol. This is indicated by the finding that the ratio of vacuolar *vs.* cytosolic LC3 particles following METH decreases dose-dependently (Figure 3(d)), which indicates a METH-induced loss of LC3 compartmentalization. Such an uncoupling between LC3 and ATG vacuoles is a novel finding in METH toxicity. In fact, so far, METH was thought to impair ATG machinery by engulfing ATG vacuoles, which become stagnant and filled with LC3. The present data show that, under METH, ATG vacuoles are impaired already in their maturation. In fact, for low METH doses, LC3 is not increased, while for higher METH doses, LC3 increases more in the cytosol than within vacuoles. It looks like that in these conditions (METH doses up to 1 *μ*M), the drive which polarizes LC3 towards the ATG machinery is weakened. At 10 *μ*M METH, there is a further drop in the ratio between vacuolar and cytosolic LC3 particles, which is likely to be due to a concomitant loss of cell ability to build organelles for toxic METH doses. This latter finding is confirmed by the fact that 10 *μ*M METH strongly increases free LC3 particles compared with the dose of 1 *μ*M, but the number of LC3-positive ATG vacuoles at 10 *μ*M is roughly a half of that counted at 1 *μ*M.

These observations, despite being unexpected, provide also novel methodological insights into ATG. In fact, when using confocal microscopy following high METH doses, there is a strong increase in LC3 immunofluorescence, which is routinely interpreted as produced by stagnant vacuoles. However, ultrastructural morphometry demonstrates that an increase in LC3 immunostaining is indeed driven by free cytosolic noncompartmentalized LC3 rather than by vacuolar LC3.

We may summarize these latter data by stating that, under METH administration, there is a loss of compartmentalization of LC3 particles within vacuoles, despite an increased amount of both LC3 particles and vacuoles per se, which represents a novel insight in ATG and METH toxicity.

This leads to reconsider the significance of densely fluorescent LC3 spots detected at confocal microscopy following METH [16, 80]. The stoichiometric counts at TEM demonstrate that a greater contribution is provided by free cytosolic LC3. This is representatively evidenced in micrograph of Figure 3(c), and it is remarked by the ratio between compartmentalized LC3 particles in ATG vacuoles and free cytosolic LC3 particles (graph of Figure 3(d)). This demonstrates a lack of METH-induced LC3 compartmentalization with a trend towards “METH-induced LC3 dispersion.” This is frankly evident for a neurotoxic dose of METH (10 *μ*M) where a loss of fine subcellular compartments take place. As discussed for Figure 1(b), this is likely to reflect a degeneration of the subcellular trim of spared cells, which organize protein trafficking, where the ability to create various cell compartments is reduced.

### 3.4. METH Alters the Amount and Placement of P20S Particles

When counting P20S-positive vacuoles, these were consistently decreased compared with controls for all METH doses (Figures 3(e) and 3(g)). These findings were reproduced when counting P20S immunogold particles, which were markedly decreased following all METH doses (ranging between 1 nM and 10 *μ*M, Figure 3(f)). Remarkably, this was replicated even for the highest dose of 10 *μ*M METH, which was shown to suppress compartmentalization of LC3 within vacuoles (Figure 3(d)). It is likely that such a discrepancy is related to a different sensitivity of P20S compared with LC3 to the effects of METH. Again, no polarization of P20S towards vacuoles was induced by METH, which left the ratio unmodified between P20S particles within vacuoles and cytosolic P20S particles compared with controls (Figure 3(h)), although the trend was different compared with LC3.

It is surprising that the effects induced by METH on the number of the ATG marker LC3 follow a different dose-response curve compared with the effects induced on the number of the UP marker P20S. In fact, in the range of 1 nM to 10 nM doses, no alterations were produced by METH in the number of LC3 immunogold particles (Figure 3(b)), while at the dose of 1 nM of METH, the reduction of P20S immunogold particles was already maximal (roughly, a half of controls, Figure 3(f)). This suggests that the biochemical pathways involved (regulating either ATG or UP) possess a different dose-response curve being the UP maximally affected already at the lowest dose of METH. Thus, the P20S protein component is markedly sensitive to doses of METH, which are likely to be in the picomolar range.

**Figure 4:**
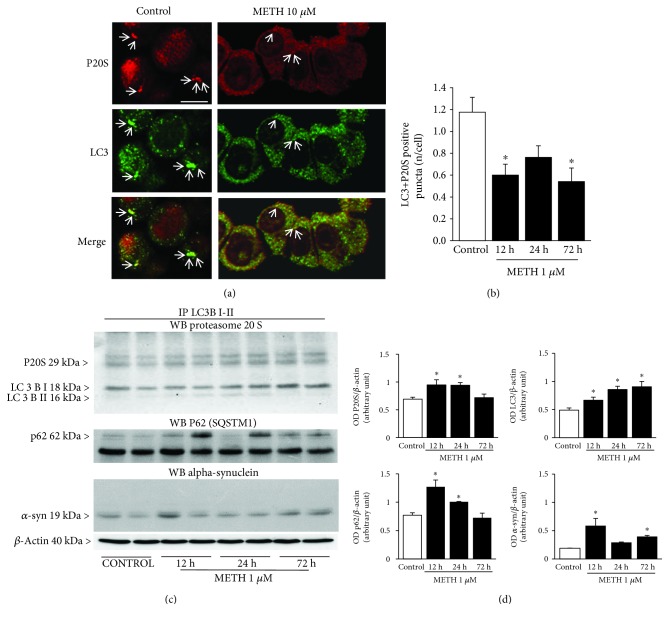
METH reduces the occurrence of the autophagoproteasome (APP) which hosts LC3, P20S, p62, and alpha-synuclein. (a) Representative immunofluorescence from controls and METH at 72 h. (b) Time-dependent graph of the number of LC3 + P20S-positive puncta per cell from control and METH at 1 *μ*M. (c) P20S, p62, and alpha-synuclein Western blotting on LC3BI-II immunoprecipitates. (d) Densitometric analysis. Values are given as the optical density detected in four separate replicates (*n* = 4). Values are given as the mean number of LC3 + P20S puncta counted in 4 slides. Error bars represent the standard error of the mean. ^∗^ *p* ≤ 0.05 vs. control. Arrows point to P20S (red fluorescence) or LC3 (green fluorescence) or merge P20S and LC3 (orange fluorescence). Scale bar = 6.6 *μ*m.

As previously discussed, the lowest dose of METH (1 nM) doubled the number of unstained vacuoles without affecting neither LC3 particles nor LC3 vacuoles. In contrast, this very same dose reduced roughly to a half both P20S particles and P20S-positive vacuoles. It is worth noting that this METH dose is sufficient to double the DA release in PC12 cells [6]. Thus, it is likely that an increased amount of free DA may already impair the P20S proteasome. This is consistent with our previous study showing that, at the dose of 1 nM of METH, P20S is already suppressed [43]. While this corresponds to a two-fold decrease in P20S-positive vacuoles (Figure 3(e)), the number of unstained METH-induced vacuoles increases by 2-fold ([6, 43]; present study in Figure 1(b)). Remarkably, UP inhibition enhances neurotransmitter release [81, 82]; in fact, proteasome inhibitors produce striatal DA release [83]. This is due to an effect of proteasome activity in the recycling of short-lived proteins from and towards the plasma membrane including DA receptors [84], which is compatible with the retromer hypothesis for unstained vacuoles expressed above [85]. It is demonstrated that increased DA stimulation disassembles the proteasome structure, and it is related to sensitization [84]. Thus, a vicious circle may establish in which METH-induced DA release alters the proteasome structure, which in turn enhances DA release. This issue opens novel avenues to study the role of protein clearing systems in determining METH-induced sensitization. Thus, the increase in DA release occurring after 1 nM METH may be due to altered proteasome levels shown in this study. This is in line with imaging of P20S following METH compared with controls at confocal microscopy. In METH-treated cells, perimembranous rings of fluorescence appear instead of the diffuse fluorescent P20S staining occurring in controls (Figure 4(a)).

The discrepancy between LC3 and P20S immunogold particles extends to the time course (Supplementary Figure 1). In fact, for longer time intervals following 10 *μ*M METH, LC3 particles increase progressively (Supplementary Figure 1a), along with LC3-positive vacuoles (Supplementary Figure 1b) with a decreasing ratio between LC3 in vacuoles and LC3 in cytosol, which is time-dependent (Supplementary Figure 1c). P20S particles and vacuoles decrease slightly (Supplementary Figure 1d and Figure 1(e), respectively). The ratio between P20S in vacuoles and P20S in cytosol was similar for all time intervals (Supplementary Figure 1f). This suggests that a loss of compartmentalization for P20S is maximal already for the lowest dose of METH.

### 3.5. METH and Autophagoproteasome (APP)

**Figure 5:**
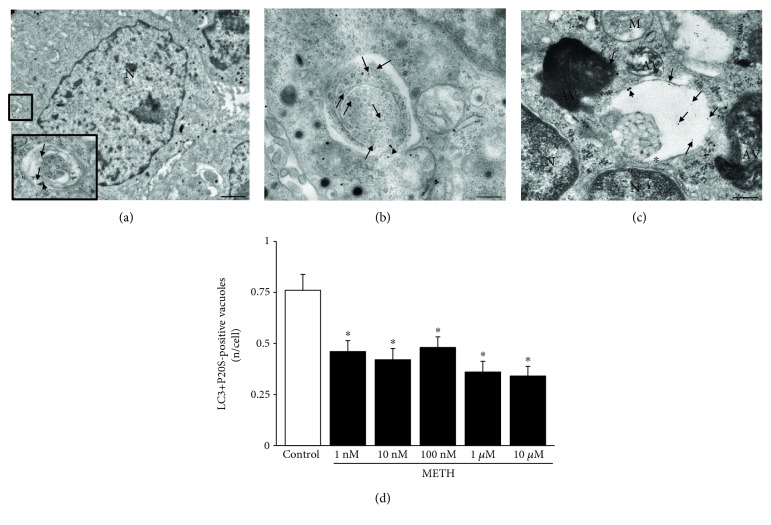
METH reduces the occurrence of autophagoproteasomes (APPs) dose-dependently. (a) Representative picture of a PC12 cell (low magnification) and an APP vacuole (high magnification). (b, c) Representative pictures of APP vacuoles stained for both LC3 (10 nm) and P20S (20 nm) immunogold particles. (d) Dose-dependent graph of the number of LC3 + P20S-positive vacuoles per cell from control and METH at 72 h. Values are given as the mean number of LC3 + P20S-positive vacuoles counted in 100 cells per group. Error bars represent the standard error of the mean. ^∗^ *p* ≤ 0.05 vs. control. Arrows point to free LC3 particles (10 nm); arrowheads point to P20S particles (20 nm); asterisk (^∗^) indicates a double membrane (b, c). N = nucleus; AV = autophagic vacuoles; M = mitochondrion. Scale bar = 220 nm.

**Figure 6:**
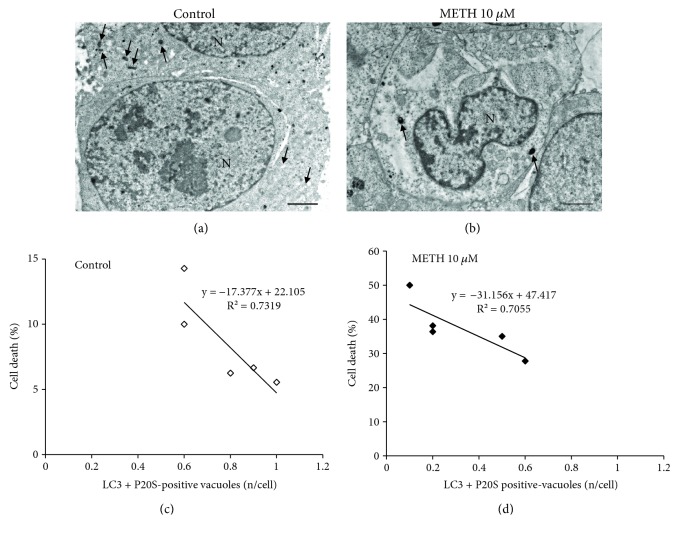
Inverse correlation between the occurrence of APP and METH-induced toxicity. (a) Representative micrograph from a control cell. (b) Representative micrograph from a cell following METH at 72 h. (c) Linear regression between the percentage of cell death and the number of LC3 + P20S-positive vacuoles in control. (d) Linear regression between the percentage of cell death and the number of LC3 + P20S-positive vacuoles following METH at 72 h. The puncta reported in the graphs ((c) white square and (d) black square) correspond to the number of grid (*n* = 5). Arrows point to vacuoles. N = nucleus. Scale bar = 1 *μ*m.

In order to document the occurrence of APP in PC12 cells and its modulation at various doses and time intervals following METH, we used confocal microscopy to document the merging between P20S and LC3 particles. As shown in representative Figure 4(a), the punctum staining for P20S and LC3 was fairly merging in baseline conditions, while only some merging could be detected also following the highest dose of METH. When we counted the amount of merging puncta detected at confocal microscopy (Figure 4(b)), these were markedly reduced following 1 *μ*M METH at each time interval (12 h, 24 h, and 72 h). Confirming the hypothesis that a chemical binding between LC3 and P20S within vacuoles exists, we carried out Western blotting on LC3BI-II immunoprecipitates from whole cell lysates. In these experimental conditions, P20S was detected along with p62 (Figures 4(c) and 4(d)). The occurrence of p62 is the key since, as recently shown by Cohen-Kaplan et al. [86], p62 is pivotal in shuttling proteasome subunits within LC3-positive autophagosomes. In line with the key role played by both ATG and proteasome to metabolize alpha-synuclein [87, 88], we checked whether these merging organelles contain alpha-synuclein. In fact, alpha-synuclein is detected here within immunoprecipitates (Figures 4(c) and 4(d)). Incidentally, these findings indicate why, in biochemical studies, the metabolism of alpha-synuclein was attributed to ATG, UP, or both, depending on the study [89–91]. The present research paper demonstrates at the morphological level the occurrence of a single organelle hosting both UP and ATG components, which recruits alpha-synuclein (Figure 4(c)). When analyzed at ultrastuctural morphometry, these merging units between P20S and LC3 appear as vacuoles owning different shapes and structures corresponding to autophagoproteasomes (APPs, Figures 4(a), 5(b), and 5(c)). It is remarkable that, according to confocal microscopy, under METH administration, a marked suppression was counted for APP for each dose of METH ranging between 1 nM and 10 *μ*M as shown in the graph of Figure 5(d). Similarly, just like it was described for the time course detected at confocal microscopy, even at TEM, APP was similarly depressed by METH at 12, 24, and 72 h (Supplementary Figure 2). The number of APP in controls (representative Figure 6(a)) and following METH (Figure 6(b)) was plotted for a regression analysis between the amount of APPs and the number of dead cells in controls (graph of Figure 6(c)) and following METH (graph of Figure 6(d)). A negative correlation was detected between cell death and the number of APPs with a slope, which was consistent in control conditions and following METH at 10 *μ*M. In fact, in both experimental conditions, cell death was lesser and lesser when APPs were more and more expressed. In controls, dead cells exceeded 10% in those samples owning only a few APPs/cell (roughly 0.6), while cell death was occluded down to 5% when APPs increased two-fold (graph of Figure 6(c)). As expected, the percentage of cell death reached almost 50% following the 10 *μ*M dose of METH, when only a few APPs were produced (roughly 0.1 per cell); in contrast, cell death was toned down to 30% in those samples in which the amount of APPs was six-fold higher (roughly 0.6 per cell, graph of Figure 6(d)).

### 3.6. The Effects of mTOR Modulation on P20S, LC3, and Autophagoproteasome Related with METH Neurotoxicity

**Figure 7:**
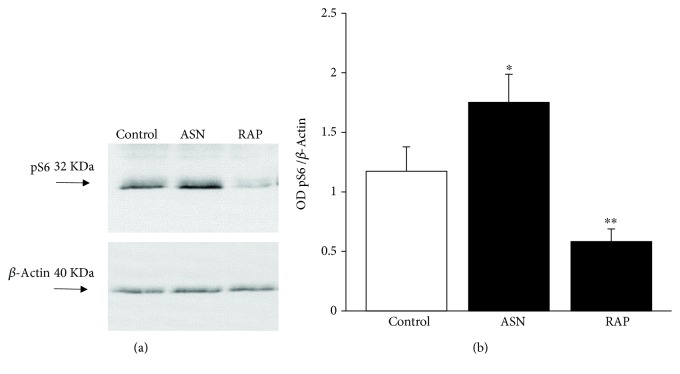
Modulation of pS6 levels underlie mTOR inhibition and activation by rapamycin and asparagine, respectively. (a) Representative SDS-PAGE immunoblotting of pS6 protein. (d) Densitometric analysis. Values are given as the optical density detected in six separate replicates (*n* = 6). Error bars represent the standard error of the mean. ^∗^ *p* ≤ 0.05 vs. control; ^∗∗^ *p* ≤ 0.05 vs. control and METH. ASN = asparagine; RAP = rapamycin.

**Figure 8:**
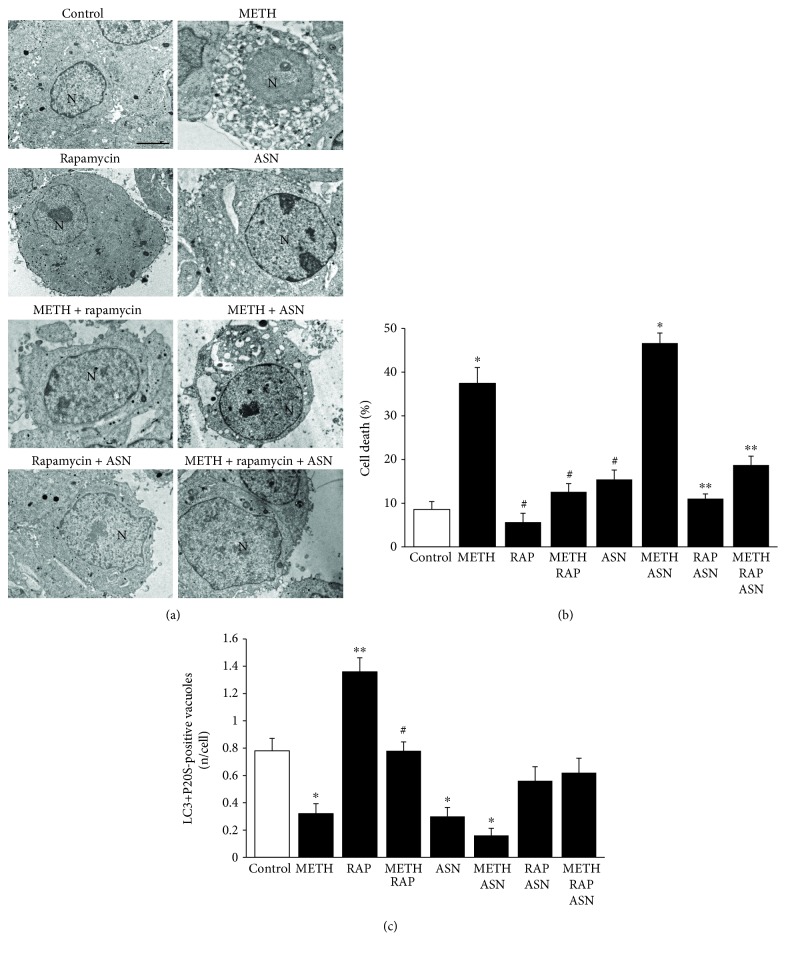
mTOR inhibition prevents cell death and rescues the amount of APPs induced by METH and the mTOR activator asparagine. (a) Representative micrographs of control and following METH 10 *μ*M, rapamycin 100 nM, and asparagine 50 mM, at 72 h. (b) Graph of the percentage of cell death in control and following METH 10 *μ*M, rapamycin 100 nM, and asparagine 50 mM, at 72 h. (c) Graph of the number of LC3 + P20S-positive vacuoles in control and following METH 10 *μ*M, rapamycin 100 nM, and asparagine 50 mM, at 72 h. For the graph in (b), values are given as the percentage of cell counted on 5 grids. For the graph in (c), values are given as the mean number of LC3 + P20S-positive vacuoles counted in 100 cells per group. Error bars represent the standard error of the mean. ^∗^ *p* ≤ 0.05 vs. control; ^∗∗^ *p* ≤ 0.05 vs. control and METH; ^#^ *p* ≤ 0.05 vs. METH. N = nucleus; ASN = asparagine; RAP = rapamycin. Scale bar = 0.5 *μ*m.

As previously published, mTOR activity finely tunes APP [47]. Therefore, in order to test in the present experiments the effects of specific doses of compounds, which are known to act either as mTOR inhibitors or activators, we measured the amount of the downstream product of mTOR activity (pS6). Asparagine is a well-known mTORC1 activator [92] while rapamycin is the gold standard mTORC1 inhibitor [93, 94]. The doses of these compounds were tested as reported in Figure 7. Asparagine at the dose of 50 mM activates mTOR while rapamycin at the dose of 100 nM inhibits mTOR as calculated by the amount of Western blotted pS6. Therefore, owning the right compounds at appropriated doses, we tested the effects of these compounds on cell death and amount of APPs. As shown in representative micrographs of Figure 8, we observed a variety of effects, which are in line with the key role of mTOR in METH toxicity and APP stimulation [80, 94, 95]. In fact, the dose of 10 *μ*M METH produces roughly 35% cell death, which was totally prevented by rapamycin (100 nM). Remarkably, rapamycin alone further reduced cell death significantly below levels found in control cells. This witnesses for the presence of a baseline inherent aberrancy of mTOR regulation in this cell line, which is reminiscent of neurodegeneration [49]. Incidentally, this is the first report showing that the gold standard inhibitor of mTOR rapamycin prevents METH toxicity. This key finding provided here as side observation is in need of a dedicated experimental project. So far, only taurine and melatonin were shown to slightly prevent METH toxicity with an indirect evidence of mTOR-mediated mechanisms [94, 96], although this was interpreted using a multifaceted hypothesis. Remarkably, recent evidence, despite not addressing directly METH neurotoxicity, demonstrated that METH-induced behavioral sensitization associates with mTOR overexpression, while rapamycin reverts such an effect [97]. Again, the stimulation of DA D1 receptors, which are key in both METH-induced toxicity and behavioral sensitization [9, 98], directly promotes mTOR activation while inhibiting autophagy [99].

The present study directly relates neuroprotection with mTOR inhibition, while showing that METH impairs autophagy. This was consolidated by the deleterious effects of asparagine. In fact, in the graph of Figure 8(b), we found that asparagine alone was slightly increasing the natural cell death occurring in control cells but it did not really increase much the amount of METH-induced cell death. When all the three compounds were coadministered, the protective effects of rapamycin prevailed, with a robust suppression of cell death occurring following METH + asparagine (graph of Figure 8). These data concerning cell death were almost mirrored by each treatment in the count of APPs. In detail, METH suppressed APPs while rapamycin increased their number almost two-fold of controls. Asparagine, as expected, depressed APPs similarly to METH, while the combination METH + asparagine produced the lowest number of APPs (3-fold less than controls). It is remarkable that rapamycin rescued the loss of APPs induced following either asparagine alone or asparagine + METH (Figure 8(c)). This strengthens the significance of the present data concerning the role of mTOR in tuning METH toxicity and APPs in a reciprocal pattern.

Here, we wish to emphasize the protective effects of mTOR inhibition on natural cell death which occurs in the PC12 cell line. In fact, these cells possess an inherent aberrancy, which is useful in understanding neuronal degeneration [49]. This is partly due to an aberrancy in DA compartmentalization and vesicle polarization, where in baseline conditions most neurotransmitter is docking to the cell membrane, making this cell line highly prone to produce massive amount of self-oxidized DA metabolites [49]. It is remarkable that upgrading APPs through mTOR inhibition erases such an inborn trend to degenerate. Since UP inhibition enhances DA release, which is related to METH toxicity, it is expected that mTOR activation, by inhibiting UP activity and compartmentalization, enhances METH-induced cell death. The present research seems to uncover the molecular determinants of inherent vulnerability of the DA-PC12 cell line, by targeting specifically mTORC1 complex dysregulation.

### 3.7. The Effects of mTOR Modulation on Unstained Vacuoles

When these experimental conditions were applied to unstained vacuoles (representative picture of Supplementary Figure 3), data obtained were quite similar to APPs, though with some exceptions. In fact, METH increases the number of unstained vacuoles, which were further increased by rapamycin alone, way more compared with LC3-positive vacuoles (roughly 20 per cell and roughly 8 per cell, respectively). This adds further information about the previous question concerning the nature of these unstained vacuoles, which turn out to be mTOR-dependent. Unexpectedly, combined administration of METH and rapamycin instead of further increasing the number of unstained vacuoles compared with rapamycin alone produces a decrease in these vacuoles (which remain higher than controls). It is likely that, in the presence of rapamycin, there is no longer an oxidative stress, which produces an altered vesicle trafficking. In fact, mTOR inhibition stimulates both the activity and the amount of the proteasome subunit, which suppresses DA release. Thus, according to the hypothesis that unstained vacuoles are due to DA release and proteasome dysfunction mutually enhancing each other, it is expected that rapamycin occludes this component. Thus, combined METH and rapamycin administration produces a number of unstained vacuoles which is still higher than controls but lower than rapamycin alone. Asparagine alone or in combination with METH decreased unstained vacuoles, which were brought up to control the levels by adding rapamycin (Supplementary Figure 3).

These data suggest that the mechanisms by which unstained vacuoles are increased are different following rapamycin compared with METH administration, since double treatment occludes this effect instead of enhancing it. This is consistent with the opposite effects on cell death, which is induced by METH and rescued by rapamycin. In contrast, APP vacuoles despite being decreased by METH were increased by rapamycin, which witnesses for a different regulation of unstained compared with APP vacuoles.

### 3.8. The Effects of mTOR Modulation on LC3

**Figure 9:**
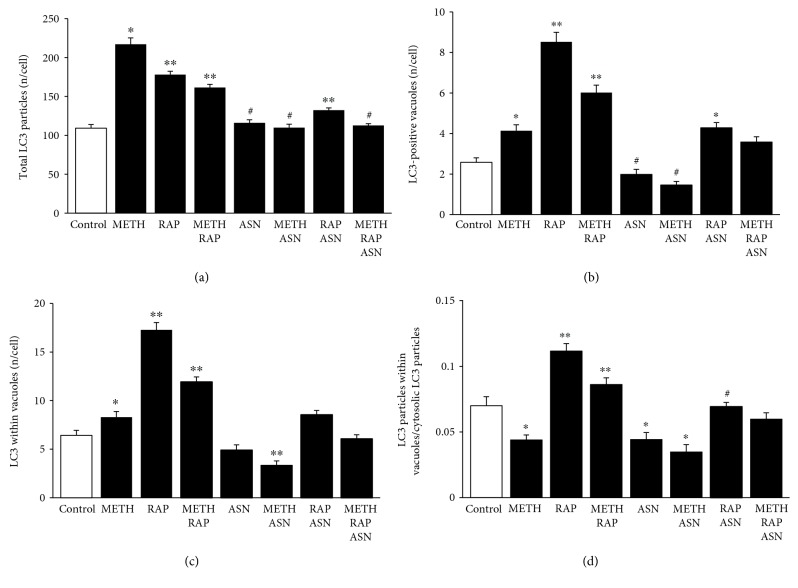
mTOR modulates the number and placement of LC3 particles. (a) The graph shows the number of total LC3 particles per cell in control, following METH 10 *μ*M, rapamycin 100 nM, and asparagine 50 mM, at 72 h. (b) The graph shows the number of LC3-positive vacuoles per cell in control, following METH 10 *μ*M, rapamycin 100 nM, and asparagine 50 mM, at 72 h. (c) The graph shows the number of LC3-positive vacuoles in control and following METH 10 *μ*M, rapamycin 100 nM, and asparagine 50 mM, at 72 h. (d) The graph shows the ratio between the number of LC3 particles within vacuoles and cytosolic LC3 particles. Values are given as the mean number of LC3 particles and vacuoles counted in 50 cells per group. Error bars represent the standard error of the mean. ^∗^ *p* ≤ 0.05 vs. control; ^∗∗^ *p* ≤ 0.05 vs. control and METH; ^#^ *p* ≤ 0.05 vs. METH.

Following mTOR inhibition by rapamycin, LC3 particles were never depressed below control values, even when METH and asparagine were combined. In these experimental conditions, compartmentalization of LC3 particles within vacuoles was dramatically enhanced by rapamycin. This mechanism was independent from the one produced by METH; in fact, despite METH 10 *μ*M was more effective than rapamycin 100 nM to increase total LC3 particles in the cell, rapamycin alone was much more powerful than METH alone in increasing LC3 within vacuoles (Figure 9(a)). Again, when rapamycin was combined with METH, a decrease of vacuolar LC3 was detected compared with rapamycin alone (Figure 9(b)). This indicates a strong compartmentalizing effect of rapamycin, which sharply contrasts with METH-induced LC3 dispersion (the generalized and nonspecific increase of LC3 promoted by METH, Figure 9(a)). The mTOR activator asparagine alone or in combination with METH further dispersed LC3 particles, since it decreased the placement of LC3 within vacuoles. This witnesses for a strong modulation by mTOR of LC3 compartmentalization (Figure 9(c)). This was further evidenced by counting the number of LC3 particles in the vacuoles versus LC3 particles within the cytosol (Figure 9(d)). In this case, METH decreases the ratio compared with controls, while rapamycin was increasing two-fold the ratio compared with controls and reverted the effects of METH. Asparagine alone was similar to METH and further suppressed the ratio when it was combined with METH.

### 3.9. The Effects of mTOR Modulation on P20S

**Figure 10:**
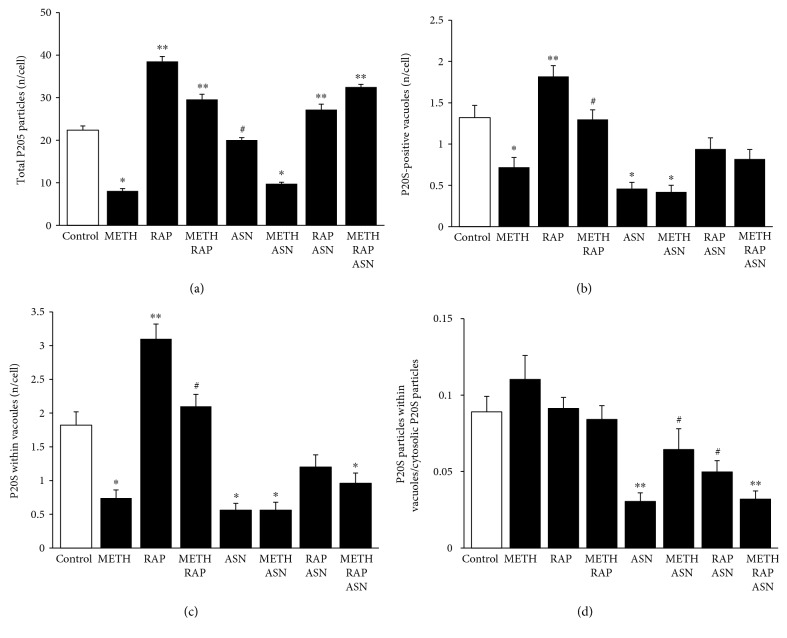
mTOR modulates the number and placement of P20S particles. (a) The graph shows the number of total P20S particles per cell in control, following METH 10 *μ*M, rapamycin 100 nM, and asparagine 50 mM, at 72 h. (b) The graph shows the number of P20S-positive vacuoles per cell in control, following METH 10 *μ*M, rapamycin 100 nM, and asparagine 50 mM, at 72 h. (c) The graph shows the number of P20S-positive vacuoles in control and following METH 10 *μ*M, rapamycin 100 nM, and asparagine 50 mM, at 72 h. (d) The graph shows the ratio between the number of P20S particles within vacuoles and cytosolic P20S particles. Values are given as the mean number of P20S particles and vacuoles counted in 50 cells per group. Error bars represent the standard error of the mean. ^∗^ *p* ≤ 0.05 vs. control; ^∗∗^ *p* ≤ 0.05 vs. control and METH; ^#^ *p* ≤ 0.05 vs. METH. ASN = asparagine; RAP = rapamycin.

The effects of mTOR inhibition were sharply contrasting with the effects of METH concerning the amount and placement of P20S particles. In fact, while METH depressed, rapamycin increased total P20S (Figure 10(a)). Moreover, rapamycin reverted the suppression induced by METH, while the effect of asparagine alone was less effective compared with METH. Combined administration of asparagine and METH did not alter the effects produced by METH alone. The effects of asparagine were antagonized by rapamycin. This was replicated by the number of P20S in the vacuoles (Figures 10(b) and 10(c)). When counting P20S-positive vacuoles or P20S in the vacuoles (Figures 10(b) and 10(c), respectively), although the general trend was similar to what is described in Figure 10(a), there was a remarkable difference concerning asparagine. In fact, the polarization of P20S within vacuoles was dramatically suppressed by this mTOR activator even when compared with METH. Moreover, the effects of asparagine on the dispersion of P20S was so powerful that even rapamycin was not able to prevent it (Figure 10(d)).

### 3.10. Correlation between Autophagoproteasomes and Cell Death

**Figure 11:**
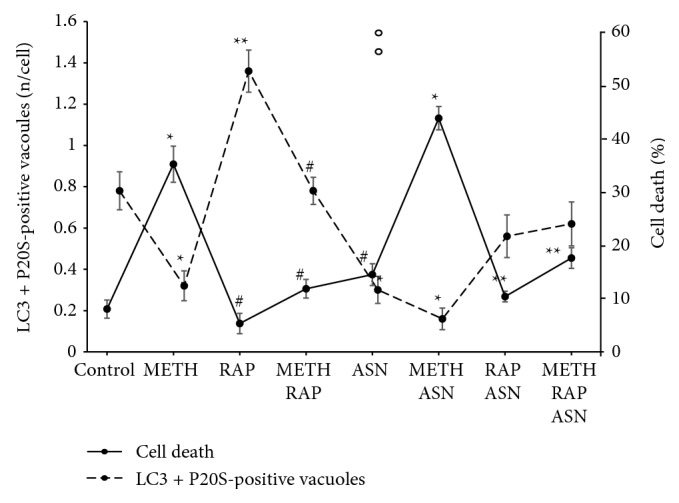
Inverse correlation between cell death and amount of APPs following mTOR modulation. The dashed line shows the amount of APPs while the continuous line shows the percentage of cell death. For each treatment, the values of the two lines produce a mirror image, which indicates a negative correlation. ^∗^ *p* ≤ 0.05 vs. control; ^∗∗^ *p* ≤ 0.05 vs. control and METH; ^#^ *p* ≤ 0.05 vs. METH. ASN = asparagine; RAP = rapamycin.

The effects of all these treatments on the amount of APPs versus the occurrence of cell death were plotted in the graph of Figure 11, which remarks for various mTOR modulators and a powerful negative correlation between the number of APPs and the number of dead cells.

In conclusions, the negative correlation which was described for APP and cell death in controls and following a 10 *μ*M dose of METH (Figure 6) was strengthened by the analysis carried out with mTOR modulators (Figure 8). This final plotting shows, at one glance, how mTOR inhibition is key for producing the merging between proteasome and autophagy to build autophagoproteasome, while it is compatible with a strong neuroprotective role exerted by such a merging organelle.

## 4. Concluding Remarks

METH administration is known to increase the number of ATG vacuoles within catecholamine-containing cells. This was originally published by Cubells et al. [22], and at first, it was suggested to produce ATG-mediated cell damage [22].

Nonetheless, in 2008, we demonstrated that the inhibition of ATG in METH-treated catecholamine cells instead of producing neuroprotection worsened METH neurotoxicity indicating a compensatory neuroprotection for ATG induction during METH toxicity, as confirmed by several studies [16, 37, 80, 100–102].

In line with this, in the present manuscript, we demonstrate that rapamycin administration fully rescues METH-induced cell death. In the present paper, apart from strengthening the concept that mTOR inhibition and ATG protect against METH toxicity, we further detail the significance of specific ATG-related structures.

It is believed that METH-induced increase in LC3 immunofluorescence is produced by an increase in LC3-positive stagnant ATG vacuoles with an impairment of the autophagy flux [37]. However, in the present study, we demonstrate that, under METH administration, there is a loss of compartmentalization of LC3 particles within vacuoles. In fact, LC3 particles increase more in the cytosol than within vacuoles, which represents a novel insight in ATG and METH toxicity.

This leads to reconsider the significance of densely fluorescent LC3 spots detected at confocal microscopy following METH, since the greatest contribution is provided by free cytosolic LC3.

In these experimental conditions, the effects of rapamycin are demonstrated to be neuroprotective against cell death while reinstating vacuolar compartmentalization of both LC3 and P20S.

It is likely that a concomitant acceleration of activity within stagnant ATG vacuoles may concur to provide neuroprotection. In fact, asparagine, which also impairs the merge between autophagosomes and lysosomes, produces a dramatic effect.

In these experimental conditions, the occurrence of ATG vacuoles is further dissected for the concomitant presence of the P20S proteasome component. It is now well established that these LC3 + P20S vacuoles contain both ATG and proteasome markers and are named “autophagoproteasomes” (APPs) [48].

In the present study, we demonstrate that LC3 + P20S-positive vacuoles (APP) represents a clearing compartment which behaves distinctly and sometimes opposite to classic ATG (LC3-positive) compartment. This specific compartment correlates with cell survival. In line with this, alpha-synuclein, which is known to buffer oxidative species [103, 104], is involved in METH toxicity, since in alpha-synuclein knockout mice, a potentiation of METH-induced nigrostriatal damage occurs [105]. The coimmunoprecipitation of alpha-synuclein within APPs found here corroborates such a neuroprotective effect. This novel organelle may counteract also impaired mitophagy during METH administration. In fact, few key steps in mitochondrial removal are carried out by proteasome components acting during early autophagosome formation [39, 106, 107].

As a proof of principle, we cannot be satisfied yet, since one might argue that a defect in ATG progression may lead ATG vacuoles not to be able to take up the proteasome component due to a failure in the p62-driven uptake of ubiquitinated proteasomes. When such an alternative explanation is consistent, then an increased amount of P20S should be measured in the cytosol. However, the number of P20S in the cytosol was decreased by METH administration, and it was further suppressed by the concomitant administration of asparagine.

Again, if the decreased amount of UP within ATG vacuoles were related to a decrease of ATG progression (impaired shuttling of P20S within ATG vacuoles), the ratio between cytosolic *vs.* vacuolar P20S should be modified by METH, while this ratio stays steady.

## Figures and Tables

**Table 1:** Sources and references for antibodies reported in the present study.

Antibodies	References
LC3 (Abcam)	[47, 51–54]
LC3 (Sigma)	[55–57]
Proteasome 20S (Abcam)	[47, 58–61]
Alpha-synuclein (BD Biosciences)	[62–66]
SQSTM1-p62 (Abcam)	[67–71]
Phospho-p70 S6 kinase (Thr421/Ser424) (Cell Signaling Technologies)	[72–76]

## Data Availability

The data used to support the findings of this study are available from the corresponding author upon request.

## Supplementary Materials

Supplementary Materials Supplementary Figures 1, 2, and 3: further evidence about various METH-induced ultrastructural alterations. In all these graphs, the dose of METH was kept constant 10 *μ*M. Supplementary Figure 1: the time dependency of METH-induced variations in LC3 and P20S particles (12 h, 24 h, and 72 h). Counts refer to whole cytosol or selectively within vacuoles. Moreover, the ratio between compartmentalized particles within vacuoles and total cytosolic particles at these time intervals is reported. Supplementary Figure 2: the time dependency of METH-induced suppression of APPs, which concerns selectively with the number of LC3 + P20S-positive vacuoles (autophagoproteasomes) in the whole cytosol. Supplementary Figure 3: the number of unstained vacuoles in the whole cytosol following various single and combined treatments with mTOR modulators.
