# Supplementary material for: mTOR Modulates Methamphetamine-Induced Toxicity through Cell Clearing Systems
Source: Oxid Med Cell Longev. 2018 Oct 29;2018:6124745. doi: 10.1155/2018/6124745 (PMC6311854; doi:10.1155/2018/6124745)
Supplement: Supplementary Materials — Supplementary Figures 1, 2, and 3: further evidence about various METH-induced ultrastructural alterations. In all these graphs, the dose of METH was kept constant 10 μM. Supplementary Figure 1: the time dependency of METH-induced variations in LC3 and P20S particles (12 h, 24 h, and 72 h). Counts refer to whole cytosol or selectively within vacuoles. Moreover, the ratio between compartmentalized particles within vacuoles and total cytosolic particles at these time intervals is reported. Supplementary Figure 2: the time dependency of METH-induced suppression of APPs, which concerns selectively with the number of LC3 + P20S-positive vacuoles (autophagoproteasomes) in the whole cytosol. Supplementary Figure 3: the number of unstained vacuoles in the whole cytosol following various single and combined treatments with mTOR modulators. [file 6124745.f1.zip › Supplementary materials/Figure Supplementary 1.docx]

**Control**

**12 h**

**24 h**

**72 h**

**METH 10 μM**

**Total LC3 particles (n/cell)**

**Control**

**12 h**

**24 h**

**72 h**

**METH 10 μM**

#

**

*

**Control**

**12 h**

**24 h**

**72 h**

**METH 10 μM**

*

*

**

**Total P20S particles (n/cell)**

**Control**

**12 h**

**24 h**

**72 h**

**METH 10 μM**

*

**a**

**Control**

**12 h**

**24 h**

**72 h**

**METH 10 μM**

*

*

**d**

**b**

**c**

**e**

**f**

**LC3 particles within vacuoles/cytosolic LC3 particles**

**P20S particles within vacuoles/cytosolic P20S particles**

**Control**

**12 h**

**24 h**

**72 h**

**METH 10 μM**

*

*
